# The Revised Mood Rhythm Instrument: A Large Multicultural Psychometric Study

**DOI:** 10.3390/jcm10030388

**Published:** 2021-01-20

**Authors:** Melissa Alves Braga de Oliveira, Euclides José de Mendonça Filho, Alicia Carissimi, Luciene Lima dos Santos Garay, Marina Scop, Denise Ruschel Bandeira, Felipe Gutiérrez Carvalho, Salina Mathur, Kristina Epifano, Ana Adan, Benicio N. Frey, Maria Paz Hidalgo

**Affiliations:** 1 Laboratório de Cronobiologia e Sono do Hospital de Clínicas de Porto Alegre, Universidade Federal do Rio Grande do Sul, Porto Alegre 90040-060, Rio Grande do Sul, Brazil; meaboliveira@gmail.com (M.A.B.d.O.); alicia.ufrgs@gmail.com (A.C.); lucienegaray@gmail.com (L.L.d.S.G.); marina.scop.medeiros@gmail.com (M.S.); gutierrezpsiq@gmail.com (F.G.C.); 2 Graduate Program in Psychiatry and Behavioral Sciences, Faculty of Medicine, Universidade Federal do Rio Grande do Sul, Porto Alegre 90040-060, Rio Grande do Sul, Brazil; 3 Graduate Program in Psychology, Universidade Federal do Rio Grande do Sul, Porto Alegre 90040-060, Rio Grande do Sul, Brazil; euclidesmendonca.f@gmail.com (E.J.d.M.F.); bandeira@ufrgs.br (D.R.B.); 4 Department of Occupational Science and Occupational Therapy, University of Toronto, Mississauga, ON L5L 1C6, Canada; salina5661@gmail.com; 5 Department of Psychology, Neuroscience & Behaviour, McMaster University, Hamilton, ON L8S 4L8, Canada; epifank@mcmaster.ca; 6 Department of Clinical Psychology and Psychobiology, School of Psychology, University of Barcelona, 08035 Barcelona, Spain; aadan@ub.edu; 7 Institute of Neurosciences, University of Barcelona, 08035 Barcelona, Spain; 8 Department of Psychiatry and Behavioural Neurosciences, McMaster University, Hamilton, ON L8N 3K7, Canada; freybn@mcmaster.ca; 9 Mood Disorders Program and Women’s Health Concerns Clinic, St. Joseph’s Healthcare, Hamilton, ON L8N 3K7, Canada

**Keywords:** mood symptoms, depressive symptoms, circadian rhythms, mood disorders, network analysis

## Abstract

Background: Recent studies with the mood rhythm instrument (MRhI) have shown that the presence of recurrent daily peaks in specific mood symptoms are significantly associated with increased risk of psychiatric disorders. Using a large sample collected in Brazil, Spain, and Canada, we aimed to analyze which MRhI items maintained good psychometric properties across cultures. As a secondary aim, we used network analysis to visualize the strength of the association between the MRhI items. Methods: Adults (*n* = 1275) between 18–60 years old from Spain (*n* = 458), Brazil (*n* = 415), and Canada (*n* = 401) completed the MRhI and the self-reporting questionnaire (SRQ-20). Psychometric analyses followed three steps: Factor analysis, item response theory, and network analysis. Results: The factor analysis indicated the retention of three factors that grouped the MRhI items into cognitive, somatic, and affective domains. The item response theory analysis suggested the exclusion of items that displayed a significant divergence in difficulty measures between countries. Finally, the network analysis revealed a structure where sleepiness plays a central role in connecting the three domains. These psychometric analyses enabled a psychometric-based refinement of the MRhI, where the 11 items with good properties across cultures were kept in a shorter, revised MRhI version (MRhI-r). Limitations: Participants were mainly university students and, as we did not conduct a formal clinical assessment, any potential correlations (beyond the validated SRQ) cannot be ascertained. Conclusions: The MRhI-r is a novel tool to investigate self-perceived rhythmicity of mood-related symptoms and behaviors, with good psychometric properties across multiple cultures.

## 1. Introduction

Several lines of research highlight the presence of alterations in circadian rhythm and sleep regulation in psychiatric and neurocognitive disorders [1,2,3,4,5,6]. Characterizing circadian functioning may optimize the management of mood disorders and promote preventive strategies in those who are at risk of developing mental disorders [7,8,9,10,11]. Biological rhythms are regulated by endogenous networks of gene activity and can be modulated by changes in the environment. Proper synchronization between light, the circadian clock and output behaviors is essential for survival [4]. Irregular exposure to light—by means of light pollution, lack of natural light during the day, night shift work, easy access to electronic devices—can disrupt circadian rhythms and sleep. Eventually, these unhealthy behaviors can lead to depressed mood [12,13]. 

Given the strong link between disturbances in biological rhythms and mood-related symptoms [5], it is important to better understand the symptoms and the phenotype of psychiatric disorders considering the temporal context of their clinical symptoms. Therefore, clinical assessment tools to evaluate the daily variability of mood are needed. We developed the mood rhythm instrument (MRhI), a 15-item self-reported questionnaire that assesses self-perceived rhythmicity of somatic, cognitive, and affective symptoms, to measure the rhythmicity of mood symptoms within the 24-h cycle [14].

The MRhI was initially created in Brazilian Portuguese [14] and was subsequently translated and validated in Spanish [15,16] and English [17] languages. Further investigation of this instrument revealed that it is not affected by recency or recall biases and it is a valid tool to investigate daily patterns of mood symptoms over 24 h [18]. Moreover, recent studies with the MRhI have shown that the presence of recurrent daily peaks in specific items are significantly associated with increased risk of psychiatric disorders, evaluated by the self-reporting questionnaire-20 [19]. Another study showed that mood-related symptoms in individuals with depressive symptoms tend to peak more frequently in the evening [20].

The main objective of the present study was to use a large dataset collected in Brazil, Spain, and Canada to provide complementary sources of validity evidence. Thus, we examined the MRhI’s factor structure, internal consistency, item fit to the measurement model, and invariance in relation to participants’ country of origin. As secondary aims, (1) network analyses were used to visualize the strength of the association of the rhythmicity of mood-related symptoms and behaviors; and (2) we investigated the association between MRhI-r and the self-reporting questionnaire-20. 

## 2. Methods

### 2.1. Sample Characterization

The study sample (*n* = 1275) was composed of 458 (35.9%) Spanish, 415 (32.5%) Brazilian, and 401 (31.4%) Canadian responders between 18 and 60 years old. Participants were recruited through snowball or convenience sampling, poster advertisements, and online research recruitment. All study participants provided written informed consent before study entry. The study was approved by the University of Barcelona (#IRB00003099), Ethics Committee of Hospital de Clínicas de Porto Alegre (#15-0539 GPPG/HCPA), and Hamilton Integrated Research Ethics Board (#2015-0619), and was conducted in accordance with the Declaration of Helsinki.

### 2.2. Mood Rhythm Instrument (MRhI)

The Spanish, Brazilian, and Canadian participants were requested to complete the MRhI. The MRhI questionnaire is composed of 15 self-reported items that are grouped into three domains: Cognitive, somatic, and affective. Each item provides a categorical question (yes/no) assessing the presence or absence of a daily peak (e.g., “Is there a specific time of the day when you have felt more sad?”). If the participants answer “yes”, they indicate on a visual analog scale (VAS) the peak time within a 24-h period (time variable, e.g., “If you answer yes, indicate below the approximate hour”). The sum of the categorical variables provided a total score, which ranged from 0 to 15, with 0 being the lowest and 15 the highest perceived rhythmicity. In short, individuals answered if there was a specific time of the day when they perceived a variety of mood-related symptoms in the last 15 days. 

### 2.3. Self-Reporting Questionnaire (SRQ-20)

The SRQ-20 consists of 20 self-reported items to screen for non-psychotic psychiatric disorders. Items have a categorical (yes/no) answer format, representing the presence or absence of a symptom. The validity, reliability, and cut-off of the SRQ-20 vary in different settings across a variety of populations [21,22,23,24]. In this study, we used the validated Brazilian Portuguese and Spanish versions and their corresponding validated screening cut-offs to detect psychiatric disorders. In the Canadian sample, the standard cut-off was applied for the English version following the developer’s suggestion [25]. Thus, scores higher than 7 were considered SRQ positive in Brazil and Canada, while scores higher than 3 were considered SRQ positive in Spain, meaning high risk for common mental disorders [26,27]. 

### 2.4. Data Analysis Procedures

Psychometric analysis of the MRhI followed three steps: Factor analysis, item response theory (IRT; Rasch analysis), and network analysis. First, we investigated the factorial structure of the inventory using exploratory factor analysis [28]. At this stage, we considered the entire sample across all countries. The polychoric correlation matrix of the data was submitted to the robust weighted least squares (WLS) estimation method with geomin oblique rotation in order to obtain results representative of the general population and appropriate correlation from categorical dichotomous variables [29]. The number of extracted factors were determined using the scree plot criteria [30] and Horn’s parallel analysis [31]. Scree plots often suggest a low number of factors and the Horn’s parallel analysis suggests a large number of factors that might overfit the model. Therefore, we evaluated the fit of a variety of dimensional models considering the following: (1) Comparative fit index and Tucker–Lewis index (TLI) > 0.9, and (2) root mean square error of approximation (RMSEA) and standardized root mean square residual (SRMR) < 0.08 [32], provided by the MPLUS package [29]. Other fit indexes such as Akaike information criterion (AIC) and Bayesian information criterion (BIC) were used to assess factorial models, with smaller values indicating a better fit [33].

Next, each dimension was analyzed separately using IRT via Rasch modeling. This framework allows for comparing participants’ parameters in relation to item properties. The IRT enables the comparison between items and people’s mood perception levels along a latent continuum [34]. Furthermore, it is possible to assess the fit of each item to the measurement model using infit mean-square statistics. This index indicates the discrepancy between the predicted patterns of response for a given item against the observed pattern. Since this model has an approximate chi-square distribution, it was possible to determine cut-off values that indicate item misfit. According to Linacre, items with infit close to one are considered perfectly fitting, items with “values above 2 distort or degrade the measurement system, and items with values between 1.5 and 2 are unproductive for measurement development, but not degrading” [35]. Since infit problems are more problematic for measurement than the outfit, items with infit above 1.5 or below 0.5, or outfit above 2.0, were deemed as not contributing adequately to the revised scale [34,35]. Subsequently, Rasch analysis was used to identify items with differential functioning (DIF) as a function of participants’ country. This analysis allowed us to verify if the scales were invariant across nationalities. Following the recommendations of Boone et al. (2014), an item difficulty contrast between the two investigated groups larger than |0.64| logits was considered evidence of a large DIF effect size. Thus, items with DIF values > |0.64| or a significant Welch test adjusted for multiple comparisons were flagged as differential functioning items. It should be highlighted that an item with DIF did not necessarily need to be removed or represent a problematic item [36]. In this case, we followed the recommendation to assess the construct-content importance of the flagged to assure that its exclusion was not detrimental to the instrument. A team of experts composed of psychiatrists, psychologists, a biomedical professional, and a medical doctor reviewed the results from the DIF and consensually agreed on the items that were redundant and could be deleted and the items that were kept in the MRhI-r.

Finally, network analysis was used to visualize the strength of the association of the MRhI items. More specifically, the nodes of the networks consisted of “mood symptoms” and the edges were the “strength of the association between the symptoms”. Gray lines in the network indicated positive partial or bivariate correlations, and the wider and more saturated the line, the stronger the correlation [37]. After item removal indicated by previous steps, a machine learning graph technique was used to visualize associations and patterns of MRhI data [38]. A graphical lasso algorithm was applied to make the network “parsimonious” and avoid the estimation of false positive edges [39]. To assess the importance of nodes in the network, we computed the node strength, which is a common metric to evaluate centrality indices of a network structure and is defined as the sum of all associations a given symptom displays with all other nodes. We also investigated the quality of the network by calculating the stability of centrality estimates and analyzing the accuracy of edge-weights using bootstrapping routines according to Epskamp, Borsboom, and Fried, 2018 [37].

The SRQ-20 was also used in our study as a tool for convergent validity. The correlation between the MRhI-r sum and SRQ-20 total scores was tested using Spearman’s correlation coefficient according to country and MRhI-r domains. Only participants who completed both questionnaires were included in this analysis (*n* = 1195). 

Data were analyzed in R Studio (R Core Team, 2017). Functions implemented by the package *psych* [40] were used to determine the number of factors; the package qgraph [41] enabled the network estimation and the package bootnet enabled the bootstrapping [37]. The factorial structure of MRhI was further investigated in Mplus and Winsteps was used for Rasch and DIF analyses. Correlation graphs were plotted using the R package ggplot2 [42].

## 3. Results

### 3.1. Factor Analysis

The inspection of the scree plot visualization indicated the retention of three factors, computation of eigenvalues of the tetrachoric correlation matrix suggested four factors with eigenvalues greater than one, and the parallel analysis determined five factors (Table 1 and Supplementary Tables S1–S3). Given that the considered criteria did not agree and the fact that parallel analyses often suggest too many factors, three to five factors were extracted, and the resulting matrices of factor loadings were inspected. The four-factor model added only 7.1% of explained shared variance compared to the three-factor model, and the five-factor model added only 5.1% of explained shared variance compared to the four-factor model. Therefore, we opted to retain three factors for subsequent analysis since it presented adequate fit indexes—χ² (63) = 115.4, *p* < 0.001; CFI = 0.98; TLI = 0.97; SRMR = 0.04; and RMSEA = 0.02—explaining 53.3% of items’ shared variance. More importantly, the three-factor model presented the highest content validity with regards to their meaning when grouped in each factor, thus proving to be the most interpretable in all solutions examined. The factor that explains most of the variance refers to the cognitive domain, followed by a factor containing somatic items, and lastly, the factor encompassing the affective domain.

The item *physical exercise* showed significant values in all three factors, despite a high percentage of variance not explained by the three-factor model (79%; Table 1). Due to its multidimensionality, this item was, therefore, excluded. Moreover, because *talking to friends* presented significant values in two of three factors and did not seem to agree in terms of meaning/construct to the other items in the same factor, this item was also excluded.

### 3.2. Rasch Analysis

IRT analysis using the Rasch modeling indicated that all the MRhI items were adequate, relative to the measuring model, presenting infit values within an appropriate range. The cognitive items presented a reliability of 0.9 and a separation reliability index of 13.5, with a mean infit of 0.99 (SD = 0.12), the somatic items presented a reliability of 0.99, a separation reliability of 20.2, and a mean infit of 0.98 (SD = 0.07), and the affective items presented a reliability of 0.89, a separation reliability of 9.8, and a mean infit of 1.00 (SD = 0.13).

**Figure 1:**
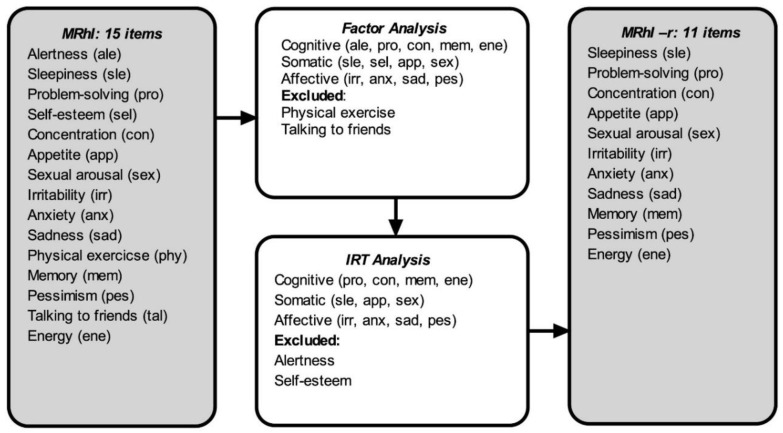
Flowchart showing the various steps in the development of the revised mood rhythm instrument (MRhI-r).

Six items exhibited considerable DIF based on their respective country of data collection (Table 2). *Alertness*, *energy, memory, irritability*, and *self-esteem* yielded a higher tendency to be perceived as rhythmic by Spain responses than in Brazil and Canada. The item *sexual arousal* demonstrated higher tendency to have a daily peak in Spain in relation to Canada. Although the items *memory* and *irritability* did not perform well in the difficulty parameters analysis, these items displayed good factor loads (0.45 and 0.44) and locations in the cognitive and affective domains, respectively. In addition, *memory* and *irritability* are key clinical features of several psychiatric disorders (e.g., memory/cognitive impairment, mixed states/mixed features); therefore, these two items were kept. Based on the clinical importance of *energy* in improving the detection and accuracy of bipolar disorder diagnosis [43], this item was kept in the final version due to its clinical relevance. Figure 1 shows the process of the reduction of the number of items from MRhI to MRhI-r and Table 3 shows the final Rasch analysis. The final version of the MRhI-r is available in the Supplemental Materials.

### 3.3. Network Analysis

Stability estimates confirmed the quality of the MRhI-r network. It displayed satisfactory accuracy indicated by small confidence intervals around the edge weights and stable strength centrality with a *CS-coefficient* of 0.28 (Figure S1).

**Figure 2:**
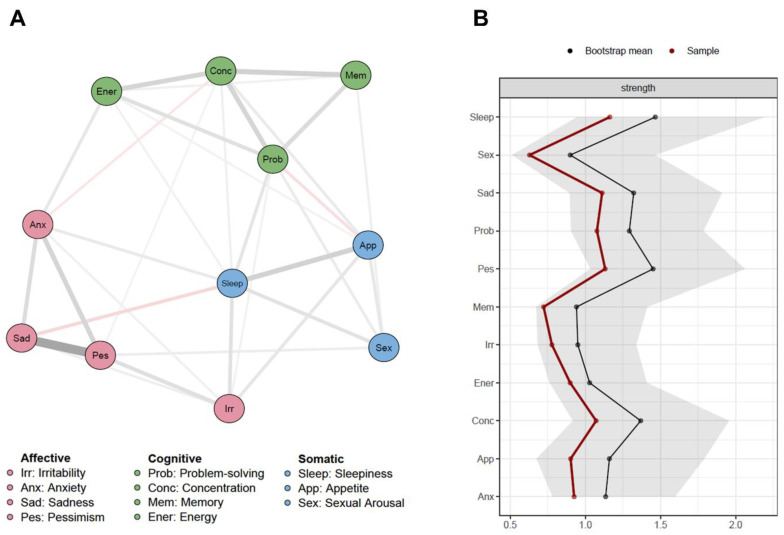
MRhI-r network. (**A**) Lasso (least absolute shrinkage and selection operator) correlations network containing the 11 items that compose the MRhI-r. Thicker lines represent stronger correlations. Gray lines stand for positive correlations and red lines for negatives correlations. (**B**) Node strength estimates (*n* = 1275), including bootstrapped 95% confidence intervals.

The intercorrelations between all MRhI items were shown through its bivariate and regularized regressions correlations (Table S4). Consistent with the factor analysis, the bivariate network illustrated how items clustered in dimensions of cognitive, somatic, and affective mood-related symptoms formed the MRhI hypothetical structure. After controlling for the mutual effects using the lasso algorithm, *sleepiness* played a central role in connecting the cognitive, somatic, and affective dimensions (Figure 2A). 

Node strength quantified how well a node was directly connected to others. For this reason, being the most central node, *sleepiness* possessed the highest value, followed by *pessimism* and *concentration* (Figure 2B).

### 3.4. Correlation with Psychiatric Disorders Screening

**Figure 3:**
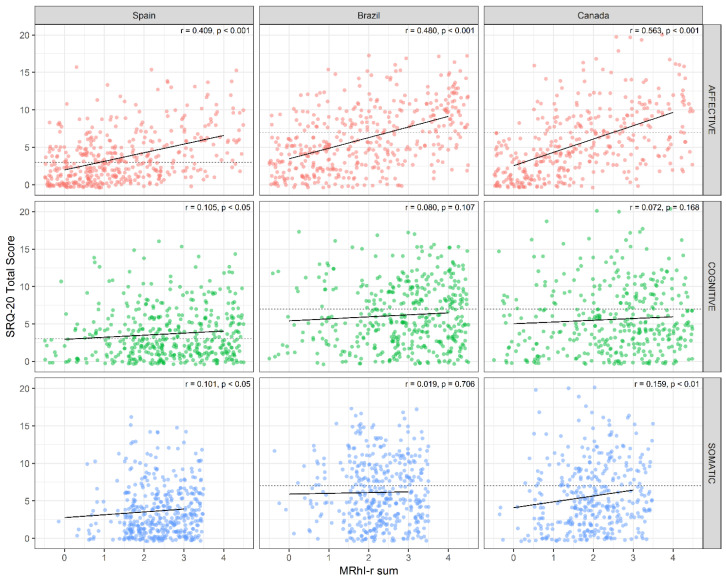
Correlations between the total sum of MRhI-r dichotomous variables (MRhI-r sum) and self-reporting questionnaire (SRQ-20) total scores (SRQ-20 score) separately for domain and country. SRQ cut-offs, which are distinct according to country, are displayed as dashed lines. Only data from participants that completed the entire SRQ were included (Spain-cognitive, *n* = 417; Spain-affective, *n* = 418; Spain-somatic, *n* = 419; Brazil-cognitive, *n* = 411; Brazil-affective, *n* = 412; Brazil-somatic, *n* = 412; Canada-cognitive, *n* = 367; Canada-affective, *n* = 367; Canada-somatic, *n* = 367). The significant correlations were in affective domains for all countries, in the cognitive domain for Spain, and in the somatic domain for Spain and Canada.

The correlation between the total sum of MRhI-r dichotomous variables and SRQ-20 total scores showed that, independent of the country, the more that individuals perceived the presence of daily peaks of mood symptoms, the higher the risk for psychiatric disorders (Figure 3), an association which was driven primarily by the affective dimension.

## 4. Discussion

In this large (N = 1275) multicenter cross-cultural study, we used multiple psychometric analyses including the MRhI’s factor structure, internal consistency, item fit to the measurement model, and its invariance in relation to participants’ country of origin. The factor analysis supported the retention of three factors, grouping the MRhI items into cognitive, somatic, and affective domains. Results from factor analysis, item response theory analysis, and clinical relevance were used to refine the MRhI into a more concise and psychometrically sound version, the MRhI-r. Finally, after controlling for the mutual effects, the network analysis showed a structure where *sleepiness* plays a central role in connecting the cognitive, somatic, and affective dimensions. 

In the development of clinical instruments measuring self-perceived outcomes that might be modulated by differences in local/cultural perspectives, it is critical to test its psychometric properties across different countries. For instance, we found in the IRT analyses that Brazilian and Canadian subjects had similar tendencies to endorse the MRhI items; in contrast, the Spanish population had a higher tendency to report daily peaks of *memory*, *irritability*, and *concentration*, and a lower tendency to report daily peaks of *energy*. We recently found that the self-perception of daily peaks of *pessimism* and *motivation to exercise* were associated with risk for psychiatric disorders in Spanish and Brazilian individuals [19]. In the present study, when we examined this association in a larger sample across three culturally diverse countries using the revised version of the MRhI, we found that the association between self-perceived daily peaks of mood symptoms and risk of psychiatric disorders was maintained and was primarily driven by affective items like *irritability*, *anxiety*, *sadness*, and *pessimism*. The affective dimension was the only domain that maintained a significant correlation with SRQ scores across all three countries. These results were consistent with another recent study in a non-clinical sample of young adults from Colombia showing that higher self-perceived mood rhythmicity of *self-esteem*, *irritability*, *anxiety*, *sadness*, and *pessimism* were associated with higher scores in the hospital anxiety and depression scale (*n* = 352) compared to individuals with lower depressive scores (*n* = 114) [20].

The use of network analysis to study psychopathological states is an innovative analytical approach that has been recently used to identify symptoms with the greatest importance in the network structure, in terms of centrality and strength of associations within the network [44,45]. This approach has been applied to identify symptoms that can predict the onset of depression [46], to distinguish individuals with and without bipolar disorder through different activation patterns of affect and physical activity [47], and to uncover specific bridge symptoms connecting two co-morbid psychiatric disorders [48]. In the present study, the network analysis was consistent with the factor analyses showing that the structure of the three dimensions was preserved. Regarding the edges, the cognitive dimension had the strongest connections, followed by the affective dimension. An interesting result from the network analysis was that *sleepiness* was positioned with high centrality, which reinforces its importance as a core construct of mood states from a self-perceived rhythmicity perspective [49]. This result is consistent with clinical studies in depression, reporting a bidirectional association between sleep disturbance and depressed mood, where insomnia has been described as a predictor or a residual symptom of depression [50,51]. Future studies applying the MRhI-r in clinical samples of individuals with major depression will allow us to deconstruct the heterogeneous phenotypes of depression from a different angle.

The present results must be considered in accordance with the limitations of our study. First, most individuals who participated in this study were young university students, so our results might not reflect the self-perceived rhythmicity of mood-related symptoms in older populations. Another limitation is that we did not conduct a formal psychological/psychiatric assessment with these individuals, so any potential clinical correlations (beyond the validated SRQ) cannot be ascertained. Currently, to address this concern, we are using the MRhI and MRhI-r in well-characterized clinical samples of individuals with major depression.

In conclusion, using multiple psychometric analyses, we were able to refine the MRhI instrument into a more psychometrically sound 11-item revised version. A better understanding of self-perceived daily peaks of mood-related symptoms may help advance the knowledge of the role of biological rhythms in mood and related disorders.

## Figures and Tables

**Table 1:** Factor analysis considering the whole sample.

	Cognitive	Somatic	Affective	*U*
Q5 Concentration	0.83 *	−0.02	−0.03	0.34
Q1 Alertness	0.64 *	−0.05	0.03	0.60
Q15 Energy	0.59 *	0.02	0.13	0.55
Q3 Problem-solving	0.53 *	0.22 *	0.01	0.54
Q12 Memory	0.45 *	0.21 *	0.04	0.62
Q11 Physical exercise	0.28 *	0.28 *	−0.26 *	0.79
Q4 Self-esteem	−0.01	0.57 *	0.29	0.48
Q7 Sexual arousal	−0.07	0.56 *	0.10	0.67
Q2 Sleepiness	0.25	0.49 *	−0.03	0.58
Q6 Appetite	0.07	0.37 *	0.08	0.79
Q14 Talking to friends	0.06	0.31 *	0.21 *	0.78
Q13 Pessimism	0.01	−0.06	0.87 *	0.27
Q10 Sadness	−0.05	0.01	0.83 *	0.32
Q9 Anxiety	0.10	0.01	0.62 *	0.55
Q8 Irritability	0.06	0.22*	0.44 *	0.65
Eigenvalues	4.90	1.89	1.19	
% variance	32.7	12.7	8.0	

* *p* < 0.05.

**Table 2:** Item difficulty, fit measures, and differential item functioning of subscales (MRhI—13 items).

		Difficulty	Infit	Difficulty Measure			Differential Item Functioning Contrast		
				SP	BR	CA	SP-BR	SP-CA	BR-CA
Cognitive	Memory	2.10	1.02	1.48	2.45	2.55	−0.97 *	−1.07 *	−0.10
	Concentration	−1.33	0.84	−1.67	−1.33	−1.01	−0.34	−0.66	−0.32
	Alertness	−0.64	1.00	−0.02	−1.39	−0.90	1.37 *	0.88 *	−0.49
	Energy	−0.16	0.94	0.71	−0.72	−0.83	1.43 *	1.54 *	0.11
	Problem-solving	0.04	0.91	−0.10	0.34	−0.10	−0.44	0.00	0.44
Somatic	Sleepiness	−3.97	1.02	−3.71	−3.60	−4.39	−0.11	0.68	0.79
	Self-esteem	2.75	0.99	3.35	2.53	2.30	0.82 *	1.05 *	0.23
	Sexual arousal	2.46	0.94	2.12	2.59	2.81	−0.47	−0.69 *	−0.22
	Appetite	−1.25	0.96	−1.63	−1.20	−0.98	−0.42	−0.64	−0.22
Affective	Irritability	−1.65	1.16	−2.16	−1.35	−1.39	−0.81 *	−0.77 *	0.04
	Pessimism	1.00	0.88	0.80	1.14	1.08	−0.34	−0.28	0.06
	Sadness	0.57	0.91	0.88	0.50	0.32	0.38	0.56	0.18
	Anxiety	0.08	1.02	0.40	−0.22	0.03	0.62	0.37	−0.25

* Welch significance test, *p* < 0.016. SP: Spain, BR: Brazil, CA: Canada.

**Table 3:** Item difficulty, fit measures, and differential item functioning of subscales (MRhI—11 items).

		Difficulty	Infit	Difficulty Measure			Differential Item Functioning Contrast		
				SP	BR	CA	SP-BR	SP-CA	BR-CA
Cognitive	Memory	2.45	1.09	1.60	2.78	3.28	−1.18 *	−1.68 *	−0.51
	Concentration	−1.87	0.97	−2.31	−1.87	−1.43	−0.44	−0.88 *	−0.45
	Energy	−0.40	1.00	0.61	−1.12	−1.19	1.73 *	1.79 *	0.06
	Problem-solving	−0.17	0.91	−0.36	0.17	−0.27	−0.53	−0.09	0.44
Somatic	Sleepiness	−3.57	1.05	−3.19	−3.26	−3.96	0.08	0.77	0.69
	Sexual arousal	4.04	1.00	4.04	3.98	4.14	0.07	−0.09	−0.16
	Appetite	−0.47	0.88	−0.57	−0.52	−0.36	−0.04	−0.21	−0.16
Affective	Irritability	−1.65	1.16	−2.16	−1.35	−1.39	−0.81 *	−0.77 *	0.04
	Pessimism	1.00	0.88	0.80	1.14	1.08	−0.34	−0.28	0.06
	Sadness	0.57	0.91	0.80	0.50	0.32	0.38	0.56	0.18
	Anxiety	0.08	1.02	0.40	−0.22	0.03	0.62	0.37	−0.25

* Welch significance test, *p* < 0.016. SP: Spain, BR: Brazil, CA: Canada.

## Supplementary Materials

The following are available online at https://www.mdpi.com/2077-0383/10/3/388/s1, the instrument can be found as the supplementary file Mood Rhythm Instrument—Revised version (MRhI-r). Figure S1: Network stability of MRhI-r items (*n* = 1275). (**A**) Edge-weight accuracy. Bootstrapped 95% confidence intervals (CI) of estimated edge-weights for the MRhI-r network are displayed as gray area. Horizontal lines represent each of the edges of the network, ordered from the edge with the highest to the one with the lowest edge-weight. The smaller the CIs, the higher the accuracy of network estimation. (**B**) Stability of strength centrality. Applying the case-dropping subset bootstrap we verified if centrality estimates remained the same with less cases. To quantify the stability, we used the *CS*-coefficient, which should not be below 0.25 according to Epskamp, Borsboom, and Fried, 2018. When the correlation after dropping a large number of participants remains high, it means that the centrality estimates in the original network can be considered stable. The *CS-coefficient* calculated was 0.28. Table S1: Eigenvalues for sample correlation matrix. Table S2: Factor analysis (four factors). Table S3: Factor analysis (five factors). Table S4: Bivariate and regularized regressions correlations of the MRhI-r items.
