# Supplementary material for: The Revised Mood Rhythm Instrument: A Large Multicultural Psychometric Study
Source: J Clin Med. 2021 Jan 20;10(3):388. doi: 10.3390/jcm10030388 (PMC7864209; doi:10.3390/jcm10030388)
Supplement: Supplementary file 1 [file jcm-10-00388-s001.zip › Supplem_Material_MRI-r_English.pdf]

UFRGS UNIVERSIDADE FEDERAL DO RIO GRANDE DO SUL

HOSPITAL DE CLÍNICAS PORTO ALEGRE RS

CRONO

MOOD RHYTHM INSTRUMENT – Revised version (MRhI-r)

Date: ____/_____/_____ **Sex:** ( )F ( )M **Age:** _______ **Subject ID:**____________

Level of education (years of schooling): _______ **Date of last period:** ____/____/_____

➔ Please read carefully the following examples before answering the questions:

Following each question, a 24-hour period is shown. Each number represents an hour of the clock. The line between the numbers represents the minutes.

Example (A): if you mark a line between 7AM and 8AM, it will represent 7:30 AM

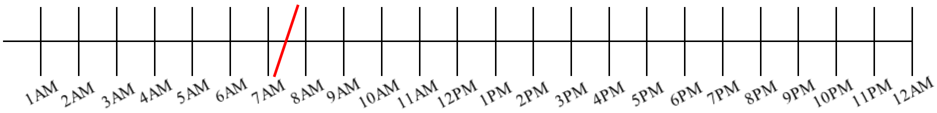

Example (B): If you mark a line on 3PM, it will represent 3:00 PM

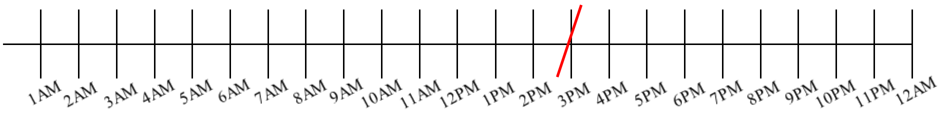

➔ Answer the following questions according to the **last 15 days**, taking into account how you have felt most of the time, on the majority of the days.

1. Is there a specific time of the day when you have felt sleepier?
( ) Yes ( ) No
If you answer yes, indicate below the approximate hour:

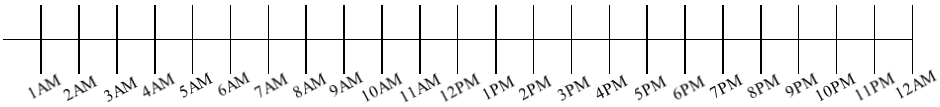

2. Is there a specific time of the day when you have felt more capable of solving daily problems?
( ) Yes ( ) No
If you answer yes, indicate below the approximate hour:

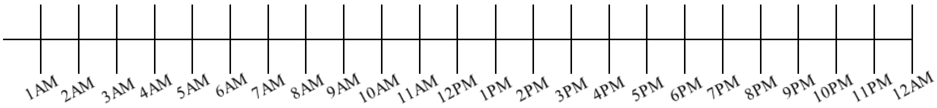

3. Is there a specific time of the day when you been able to concentrate better?
( ) Yes ( ) No
If you answer yes, indicate below the approximate hour:

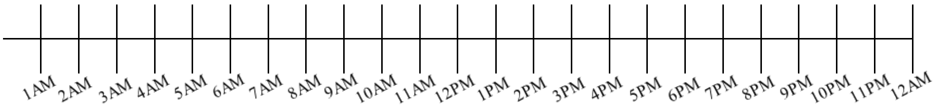

4. Is there a specific time of the day when you have had an increased appetite?
( ) Yes ( ) No
If you answer yes, indicate below the approximate hour:

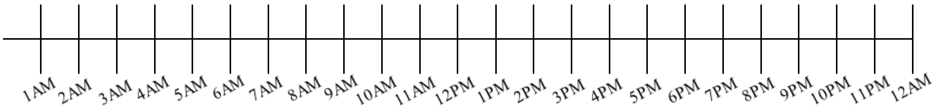

UFRGS UNIVERSIDADE FEDERAL DO RIO GRANDE DO SUL — HOSPITAL DE CLÍNICAS PORTO ALEGRE RS — CRONOSONO

MOOD RHYTHM INSTRUMENT – Revised version (MRhI-r)

5. Is there a specific time of the day when your libido (sexual arousal) has been higher?
( ) Yes ( ) No
If you answer yes, indicate below the approximate hour:

1AM 2AM 3AM 4AM 5AM 6AM 7AM 8AM 9AM 10AM 11AM 12PM 1PM 2PM 3PM 4PM 5PM 6PM 7PM 8PM 9PM 10PM 11PM 12AM

6. Is there a specific time of the day when you have felt more irritable?
( ) Yes ( ) No
If you answer yes, indicate below the approximate hour:

1AM 2AM 3AM 4AM 5AM 6AM 7AM 8AM 9AM 10AM 11AM 12PM 1PM 2PM 3PM 4PM 5PM 6PM 7PM 8PM 9PM 10PM 11PM 12AM

7. Is there a specific time of the day when you have felt more anxious?
( ) Yes ( ) No
If you answer yes, indicate below the approximate hour:

1AM 2AM 3AM 4AM 5AM 6AM 7AM 8AM 9AM 10AM 11AM 12PM 1PM 2PM 3PM 4PM 5PM 6PM 7PM 8PM 9PM 10PM 11PM 12AM

8. Is there a specific time of the day when you have felt more sad?
( ) Yes ( ) No
If you answer yes, indicate below the approximate hour:

1AM 2AM 3AM 4AM 5AM 6AM 7AM 8AM 9AM 10AM 11AM 12PM 1PM 2PM 3PM 4PM 5PM 6PM 7PM 8PM 9PM 10PM 11PM 12AM

9. Is there a specific time of the day when your memory has been better?
( ) Yes ( ) No
If you answer yes, indicate below the approximate hour:

1AM 2AM 3AM 4AM 5AM 6AM 7AM 8AM 9AM 10AM 11AM 12PM 1PM 2PM 3PM 4PM 5PM 6PM 7PM 8PM 9PM 10PM 11PM 12AM

10. Is there a specific time of the day when you have been more pessimistic?
( ) yes ( ) No
If you answer yes, indicate below the approximate hour:

1AM 2AM 3AM 4AM 5AM 6AM 7AM 8AM 9AM 10AM 11AM 12PM 1PM 2PM 3PM 4PM 5PM 6PM 7PM 8PM 9PM 10PM 11PM 12AM

11. Is there a specific time of day when you have had more energy?
( ) yes ( ) No
If you answer yes, indicate below the approximate hour:

1AM 2AM 3AM 4AM 5AM 6AM 7AM 8AM 9AM 10AM 11AM 12PM 1PM 2PM 3PM 4PM 5PM 6PM 7PM 8PM 9PM 10PM 11PM 12AM
