# Supplementary material for: The Revised Mood Rhythm Instrument: A Large Multicultural Psychometric Study
Source: J Clin Med. 2021 Jan 20;10(3):388. doi: 10.3390/jcm10030388 (PMC7864209; doi:10.3390/jcm10030388)
Supplement: Supplementary file 1 [file jcm-10-00388-s001.zip › jcm-949506 supple figure final.docx]

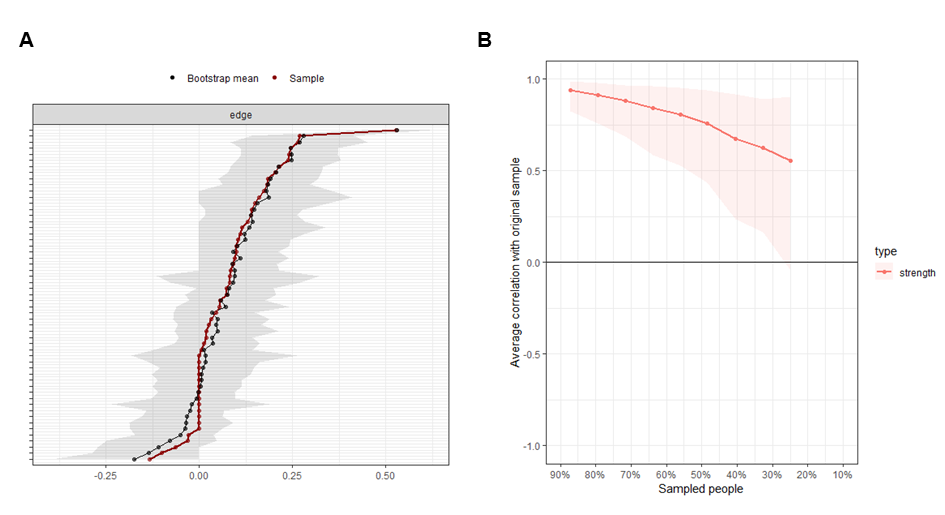


**Figure S1.** Network stability of MRhI-r items (*n* = 1275). (**A**) Edge-weight accuracy. Bootstrapped 95% confidence intervals (CI) of estimated edge-weights for the MRhI-r network are displayed as gray area. Horizontal lines represent each of the edges of the network, ordered from the edge with the highest to the one with the lowest edge-weight. The smaller the CIs, the higher the accuracy of network estimation. (**B**) Stability of strength centrality. Applying the case-dropping subset bootstrap we verified if centrality estimates remained the same with less cases. To quantify the stability, we used the CS-coefficient, which should not be below 0.25 according to Epskamp, Borsboom, and Fried, 2018. When the correlation after dropping a large number of participants remains high, it means that the centrality estimates in the original network can be considered stable. The CS-coefficient calculated was 0.28.
