# Supplementary material for: The Revised Mood Rhythm Instrument: A Large Multicultural Psychometric Study
Source: J Clin Med. 2021 Jan 20;10(3):388. doi: 10.3390/jcm10030388 (PMC7864209; doi:10.3390/jcm10030388)
Supplement: Supplementary file 1 [file jcm-10-00388-s001.zip › jcm-949506 supple tables final.docx]

**Table S1.** Eigenvalues for sample correlation matrix.

| **Factors to retain** | **Eigenvalue** | **Proportional explained variance** | **Cumulative explained variance** |
| --- | --- | --- | --- |
| 1 | 4.902 | 32.7% | 32.7% |
| 2 | 1.899 | 12.7% | 45.3% |
| 3 | 1.193 | 8.0% | 53.3% |
| 4 | 1.064 | 7.1% | 60.4% |
| 5 | 0.878 | 5.9% | 66.2% |
| 6 | 0.759 | 5.1% | 71.3% |
| 7 | 0.684 | 4.6% | 75.9% |
| 8 | 0.621 | 4.1% | 80.0% |
| 9 | 0.582 | 3.9% | 83.9% |
| 10 | 0.570 | 3.8% | 87.7% |
| 11 | 0.464 | 3.1% | 90.8% |
| 12 | 0.445 | 3.0% | 93.7% |
| 13 | 0.395 | 2.6% | 96.4% |
| 14 | 0.293 | 2.0% | 98.3% |
| 15 | 0.251 | 1.7% | 100.0% |

**Table S2.** Factor analysis (four factors).

|  | **F1** | **F2** | **F3** | **F4** | ***U*** |
| --- | --- | --- | --- | --- | --- |
| Q2 Sleepiness | 0.87 * | 0.05 | −0.04 | −0.08 | 0.23 |
| Q6 Appetite | 0.49 * | −0.02 | 0.06 | 0.08 | 0.72 |
| Q5 Concentration | −0.04 | 0.83 * | −0.01 | −0.00 | 0.36 |
| Q1 Alertness | 0.10 | 0.62 * | 0.08 | −0.20 * | 0.55 |
| Q15 Energy | 0.05 | 0.58 * | 0.15 * | −0.01 | 0.56 |
| Q3 Problem-solving | 0.04 | 0.58 * | −0.03 | 0.23 * | 0.52 |
| Q12 Memory | −0.09 | 0.56 * | −0.01 | 0.32 * | 0.55 |
| Q13 Pessimism | 0.01 | 0.01 | 0.84 * | 0.01 | 0.29 |
| Q10 Sadness | −0.06 | −0.01 | 0.80 * | 0.14 | 0.30 |
| Q9 Anxiety | 0.15 | 0.06 | 0.63 * | −0.06 | 0.53 |
| Q8 Irritability | 0.31 * | 0.01 | 0.41 * | 0.07 | 0.63 |
| Q11 Physical Exercise | 0.25 * | 0.26 * | −0.27 * | 0.11 | 0.79 |
| Q4 Self-esteem | 0.32 | 0.04 | 0.19 * | 0.45 * | 0.49 |
| Q7 Sexual Arousal | 0.37 * | −0.05 | 0.02 | 0.38 * | 0.69 |
| Q14 Talking to Friends | 0.04 | 0.12 | 0.13 | 0.40 * | 0.72 |

**p* < 0.05.

**Table S3.** Factor analysis (five factors).

|  | **F1** | **F2** | **F3** | **F4** | **F5** | ***U*** |
| --- | --- | --- | --- | --- | --- | --- |
| Q5 Concentration | 0.80 * | 0.03 | −0.04 | −0.00 | 0.04 | 0.35 |
| Q1 Alertness | 0.60 * | 0.13 | 0.06 | −0.02 | −0.12 | 0.58 |
| Q15 Energy | 0.56 * | 0.10 | 0.12* | −0.01 | 0.06 | 0.55 |
| Q3 Problem-solving | 0.56 * | −0.08 | −0.01 | 0.29* | 0.02 | 0.52 |
| Q12 Memory | 0.52 * | −0.18 | 0.02 | 0.30* | 0.03 | 0.57 |
| Q2 Sleepiness | 0.10 | 0.64 * | −0.03 | 0.26 | −0.05 | 0.34 |
| Q6 Appetite | −0.04 | 0.55 * | 0.04 | 0.05 | 0.20 | 0.64 |
| Q13 Pessimism | 0.02 | 0.02 | 0.85 * | 0.02 | −0.06 | 0.28 |
| Q10 Sadness | −0.01 | −0.08 | 0.82 * | 0.10 | −0.02 | 0.30 |
| Q9 Anxiety | 0.07 | 0.18* | 0.62 * | −0.05 | 0.01 | 0.53 |
| Q8 Irritability | 0.01 | 0.33* | 0.41 * | 0.01 | 0.15 | 0.61 |
| Q4 Self-esteem | 0.02 | 0.04 | 0.25 * | 0.57 * | 0.06 | 0.46 |
| Q7 Sexual Arousal | −0.06 | 0.12 | 0.07 | 0.49 * | 0.05 | 0.68 |
| Q11 Physical Exercise | 0.25* | 0.09 | −0.24 * | 0.31 * | −0.10 | 0.76 |
| Q14 Talking to Friends | 0.04 | 0.00 | −0.00 | 0.02 | 0.97 * | 0.03 |

**p* < 0.05.

**Table S4.** Bivariate and regularized regressions correlations of the MRhI-r items.

|  | **Problem-solving** | **Concentration** | **Memory** | **Energy** | **Irritability** | **Anxiety** | **Sadness** | **Pessimism** | **Sleepiness** | **Appetite** | **Sexual Arousal** |
| --- | --- | --- | --- | --- | --- | --- | --- | --- | --- | --- | --- |
| Problem-solving |  | 0.26 | 0.21 | 0.19 | 0.1 | 0.05 | 0.02 | −0.07 | 0.16 | −0.17 | 0.15 |
| Concentration | 0.51 |  | 0.28 | 0.25 | 0.00 | −0.15 | 0.01 | 0.11 | 0.10 | 0.15 | −0.06 |
| Memory | 0.46 | 0.49 |  | 0.09 | −0.01 | 0.05 | 0.03 | 0.01 | −0.04 | 0.01 | 0.11 |
| Energy | 0.45 | 0.49 | 0.38 |  | −0.02 | 0.15 | 0.07 | −0.01 | 0.09 | 0.09 | −0.07 |
| Irritability | 0.27 | 0.25 | 0.20 | 0.26 |  | 0.08 | 0.10 | 0.20 | 0.19 | 0.17 | −0.04 |
| Anxiety | 0.23 | 0.16 | 0.21 | 0.33 | 0.40 |  | 0.21 | 0.26 | 0.15 | 0.06 | −0.06 |
| Sadness | 0.19 | 0.20 | 0.22 | 0.27 | 0.40 | 0.52 |  | 0.53 | −0.20 | 0.07 | 0.05 |
| Pessimism | 0.20 | 0.24 | 0.22 | 0.26 | 0.46 | 0.55 | 0.71 |  | 0.02 | −0.12 | 0.14 |
| Sleepiness | 0.37 | 0.35 | 0.23 | 0.34 | 0.36 | 0.28 | 0.08 | 0.18 |  | 0.27 | 0.19 |
| Appetite | 0.14 | 0.28 | 0.17 | 0.26 | 0.32 | 0.22 | 0.16 | 0.15 | 0.42 |  | 0.17 |
| Sexual Arousal | 0.28 | 0.20 | 0.25 | 0.16 | 0.20 | 0.18 | 0.21 | 0.26 | 0.33 | 0.28 |  |
| Note: Values below the diagonal represent bivariate tetrachoric correlations (white); values above indicate partial correlations based on regularized regressions (shaded). | | | | | | | | | | | |
